# Antiquity of Leprosy

**Published:** 1912-12

**Authors:** 


ANTIQUITY OF LEPROSY. In *Bulletin No. 6 of the Archaeological Survey of Nubia,* Professor Elliot Smith and Dr. D. Derry describe and figure a case which shows all the typic lesions of leprosy. The subject of the disease was found in a Nubian cemetery assigned to an early Christian century. Although the tissues are at least 1,600 years old they cut and stain perfectly, but so far no leprosy bacilli have been found in them, although various forms of cocci are seen in abundance. The authors have had many opportunities of examining the remains of many thousands of ancient Nubians—covering a period of at least 6,000 years, yet this is the first instance they have seen suggestive of leprosy. No certain signs of syphilis have been found, but typic examples of tuberculosis do occur although not abundantly.

